# Sustained Heatwaves Reshape the Phytoplankton–Zooplankton Community Structure in Freshwater Ecosystems: A Case Study of Shengjin Lake

**DOI:** 10.1002/ece3.72460

**Published:** 2025-11-07

**Authors:** Lingli Jiang, Mengfan Sun, Zhongze Zhou, Yutao Wang

**Affiliations:** ^1^ School of Resources and Environmental Engineering Anhui University Hefei China; ^2^ Anhui Shengjin Lake Wetland Ecology National Long‐Term Scientific Research Base Dongzhi China; ^3^ School of Chemistry and Chemical Engineering Anhui University Hefei China

**Keywords:** keystone species, plankton, sustained heatwaves, water ecosystem

## Abstract

Extreme climate events caused by global climate change pose a huge threat to freshwater ecosystems. As the frequency and intensity of heatwaves increase, sustained heatwaves may increase their certainty in ecosystems, which may affect phytoplankton and zooplankton, the key components of freshwater ecosystems. Sustained heatwaves can lead to shifts in plankton community structure, driven by changes in immigration patterns and life history strategies, potentially resulting in altered functional characteristics of phytoplankton and zooplankton in aquatic ecosystems. However, research on the effects of sustained heatwaves on plankton has mostly been conducted in laboratories, with less data from studies in natural water bodies. Therefore, in order to understand this ecological process in natural lakes, this study investigated the phytoplankton and zooplankton in Shengjin Lake during the summer heatwave in 2022. The results show that sustained heatwaves significantly alter the composition of plankton communities: Cyanophyta dominated during the heatwave period, while Bacillariophyta replaced Cyanophyta as the dominant group during the heatwave acclimation period. For zooplankton, the community shifted from being dominated by Rotifera to being dominated by Cladocera and Copepoda. Water temperature is a key driving factor affecting plankton communities, with contribution rates of 33.83% and 38.70% to the changes in zooplankton and phytoplankton communities, respectively. The connectivity of plankton co‐occurrence networks and keystone species change dynamically with heatwaves. Sustained heatwaves can exert thermal selective pressure on plankton communities, favoring the survival of individuals or species with stronger high‐temperature tolerance and potentially inducing acclimation or other adaptive responses. These results provide data support for studying the impact of sustained heatwaves on plankton communities and also provide a new perspective for the management and protection of freshwater ecosystems under climate change.

## Graphical abstract

The sustained heatwave has shaped the plankton community structure and led to high‐temperature domestication of some of the plankton. Plankton communities have the ability to resist heatwave stress. The sustained heatwave has altered the interrelationships between plankton.

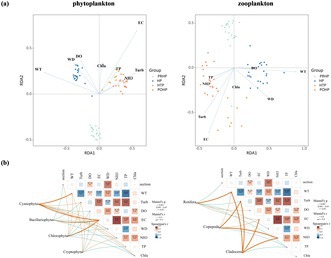

## 1 Introduction

Heatwaves are generally considered a prolonged period of hot weather that lasts for several days to weeks (Shi et al. 2021). The underlying cause of the frequency of heatwaves is climate change and global warming (Barbier et al. 2018; Park et al. 2023; Wang et al. 2020). As the global warming trend continues, the frequency and intensity of extreme heat, precipitation, droughts, and tropical cyclones increase (Dupoué et al. 2020). Heatwaves and droughts, in particular, are not only more likely to occur simultaneously, but also last longer, start earlier and end later. The ecological impacts of long‐lived heatwaves (lasting more than 5 days) are the most prominent (Yang et al. 2024, 2021).

The disturbance of extreme heatwaves to ecosystems has become the focus of global attention. Several studies have shown that extreme heatwave and drought events associated with global warming can lead to changes in the balance between photosynthesis and respiration in ecosystems, which in turn affects carbon cycling processes, and that high temperatures are among the most damaging factors for crop growth and human health (An et al. 2022; Anderson and Bell 2011; Siebers et al. 2015). In addition, in freshwater ecosystems, increased water temperatures due to high‐temperature heatwave events may alter their ecological processes, affecting their food web structure and community structure (Hermann et al. 2024). Experimental and theoretical studies have demonstrated that higher water temperature accelerates the metabolism of species, elevates the rate of energy transfer, and can lead to a simpler food web structure (Béjaoui‐Omri et al. 2014; Zambie et al. 2024). The change of the original environment due to the water temperature increase is expected to also change the community structure of aquatic organisms and the stability of aquatic ecosystems (Lv et al. 2024).

Plankton are key components of freshwater lake ecosystems and they are sensitive to environmental changes (Hays et al. 2005). Plankton is mainly composed of phytoplankton and zooplankton, and phytoplankton, as the primary producer in the lake food web, is the main source of food for lake organisms, while zooplankton is an important link between primary producers and lake macrofauna. Thus, the top‐down and bottom‐up effects between zooplankton and phytoplankton are the basis of the aquatic food web (Pineda et al. 2020; Simons et al. 2006). In recent years, studies have shown that elevated water temperature has always been a key factor affecting plankton communities. Higher water temperature is favorable for the proliferation of Cyanophyta, which causes lake blooms, whereas, higher water temperature inhibits the growth of diatoms such as *Thalassiosira* sp. and *Nitzschia closterium* (Daufresne et al. 2009; Hunt 2007). The increase in water temperature caused by heatwaves leads to heat acclimation in some plankton. That is, when zooplankton are exposed to a gradually warming environment for long or short periods, they can adapt to high‐temperature conditions through long‐term adaptation or short‐term phenotypic plasticity (Cavalheri et al. 2018; Pang et al. 2025). For example, under the background of global warming, *Daphnia longispina* has evolved a survival‐prioritized reproductive strategy characterized by “high metabolic maintenance‐low growth‐high heat resistance” (Ruiz and Kainz 2025). In the laboratory, *Daphnia pulex* exhibits short‐term phenotypic plasticity after high‐temperature induction. (Pansch et al. 2018).

In addition, the increase in water temperature caused by high‐temperature heatwaves impacts the interactions between plankton species in lake ecosystems (Caracciolo et al. 2022; Lewandowska et al. 2014). In aquatic ecosystems, predator–prey interactions are critical for maintaining the aquatic food web's stability (Kiene et al. 2024). Increased abundance of large zooplankton under the influence of high temperatures and heatwaves can intensify the pressure on small Rotifera as well as edible algae for predation and food competition (Pu et al. 2017). In addition to this, there is competition and mutually beneficial symbiosis between plankton. Ecological processes in nitrogen‐fixing Cyanophyta reduce the power of light, a metabolic capacity that is beneficial to autotrophic photosynthetic eukaryotes *Euglena pisciformis, Trachelomonas* sp. and *Phacus* sp. under dark conditions (Markensten et al. 2010).

However, current studies mostly focus on the responses to short‐term heatwaves or single taxa, and are largely confined to laboratory research (Huỳnh et al. 2024; Soulié et al. 2023; Zhan et al. 2024, 2023). There is a dearth of knowledge regarding how long‐lived heatwaves, which persist for several weeks in natural ecosystems, systematically reshape the community structures of phytoplankton and zooplankton. Furthermore, research on plankton interactions is mostly based on single relationships (such as predation), while interactions between plankton also include mutualism and parasitism (García‐Oliva and Wirtz 2025; Venegas et al. 2025). These comprehensive interactions are difficult to directly observe at the community level. Co‐occurrence networks have been widely used to explore potential relationships between microbial taxa, and this method is now also applied to analyze plankton interactions (Barberán et al. 2012). The complexity of phytoplankton–zooplankton networks can be characterized by topological features based on the results of CoNet analysis, and keystone species in the network play a crucial role in maintaining community stability (Barberán et al. 2012; Berry and Widder 2014). How heatwaves affect phytoplankton–zooplankton networks remains unclear.

From June to August 2022, affected by the combined effects of the La Niña event, the westward extension of the Western Pacific Subtropical High, and the mid‐latitude continental high, East China suffered a persistent extreme heatwave lasting more than 50 days (Chen et al. 2023; Zhou et al. 2024). Shengjin Lake, a typical freshwater lake in the middle and lower reaches of the Yangtze River, was also within the affected area, providing a research basis for exploring the impact of long‐lived heatwaves on freshwater ecosystems. For such long‐lived heatwaves that persist for several weeks, we refer to them as “sustained heatwaves”.

This study takes Shengjin Lake, which experienced a sustained heatwave in 2022, as the research object. We conducted plankton sampling in Shengjin Lake in May, July, August, and September 2022, aiming to clarify the impact mechanism of sustained heatwaves on plankton in freshwater lakes. In this study, we hypothesized that a sustained heatwave will (1) alter phytoplankton and zooplankton community structure; (2) change phytoplankton and zooplankton interactions; (3) shape keystone species in the plankton community; (4) alter community stability.

## 2 Materials and Methods

### 2.1 Study Sites and Sampling Campaign

**FIGURE 1:**
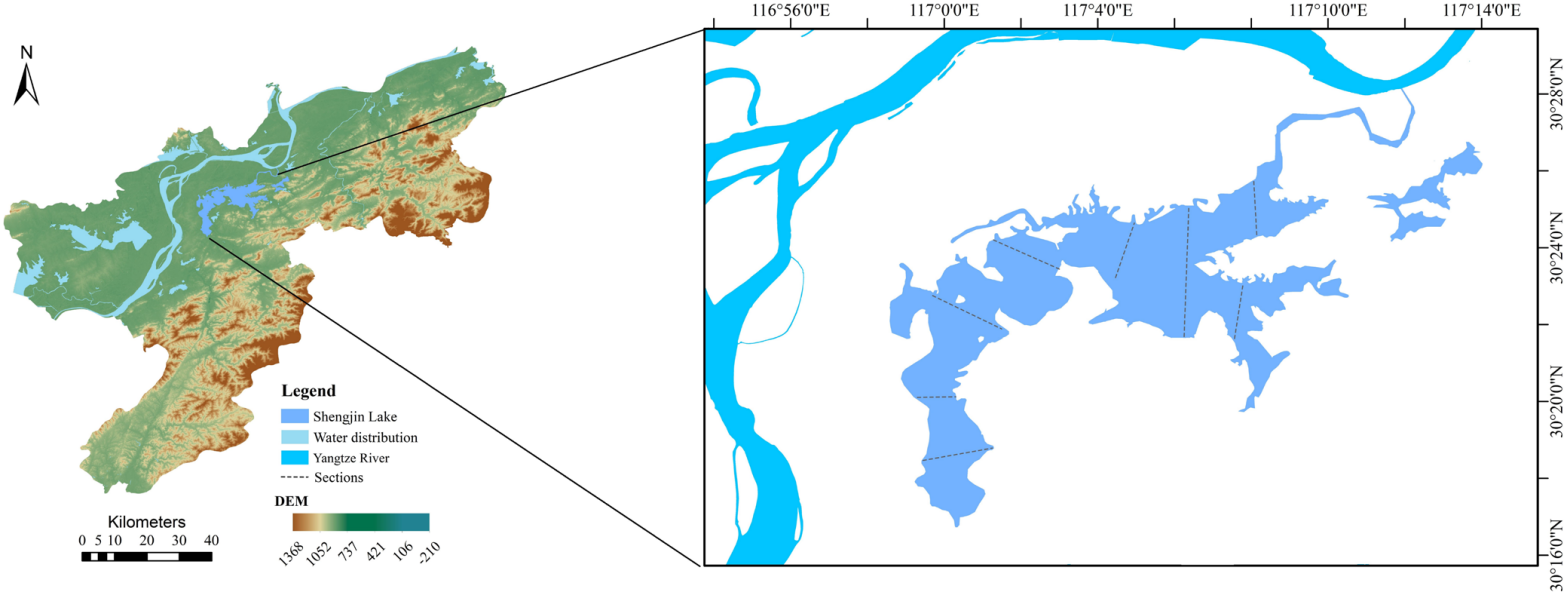
Map of the study area and sampling sections.

**FIGURE 2:**
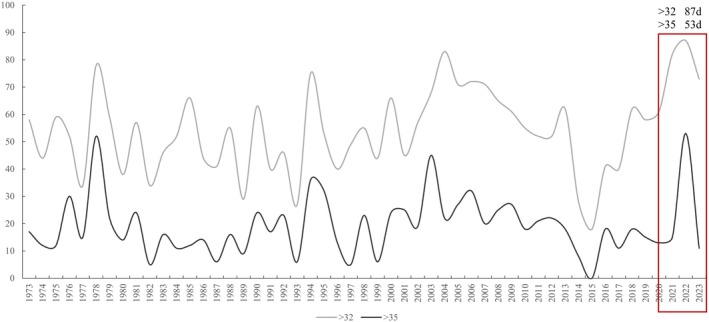
Summary of high temperature weather in Shengjin Lake from 1973 to 2023. The graph counts the number of days each year when the maximum temperature exceeds 32°C (gray line) and 35°C (black line) respectively. In 2022, there were 87 days of more than 32°C and 53 days of weather greater than 35°C.

Shengjin Lake (30°15′ N–30°28′ N, 116°58′ E–117°14′ E) borders the south bank of the Yangtze River and is located in Chizhou City, South Anhui Province, and consists of three lakes: the upper lake, the middle lake, and the lower lake (Figure 1). The upper lake and the middle lake are separated by a small road mouth bridge barrier, and the lower lake, also called Huangpen Lake, is connected to the Yangtze River. The lake area has a flatwater period (March–June, October–November), an abundant water period (July–September), and a dry water period (December–February). The water level drops rapidly in the dry water period, forming a large area of marshes and mudflats. The information of Chizhou City Water Resource Bulletin of 2013 indicates that, during the dry water period, Shengjin Lake's average level is 1.8 × 10^8^ m^3^, while in the abundant water period, it is 3.5 × 10^9^ m^3^. The sustained heatwave that hit Shengjin Lake in 2022 was the longest‐lasting in the past four decades (Figure 2).

In order to study the effects of sustained heatwaves on the plankton community, we collected water samples in May (pre‐heatwave period, PRHP), July (heatwave period, HP), August (heatwave taming period, HTP), and September (post‐heatwave period, POHP). According to the lake morphology characteristics of Shengjin Lake, we set up eight sections in the lake area and collected 1–3 samples per section with a total of 73 samples collected during the survey.

### 2.2 Data Collection

In May, July, August, and September 2022, we collected water samples from Shengjin Lake. We measured water temperature (WT), dissolved oxygen (DO), and electrical conductivity (EC) in situ using the Hach HQ40d portable multimeter, and turbidity (Turb) with the Hach 2100Q. Utilizing the Secchi disk in situ, we measured secchi depth (SD) and water depth (WD). At each sampling point, we collected water samples in pp. plastic bottles and brought them to the laboratory within 48 h to measure the nutrient concentrations, including ammonia (NH_3_), total nitrogen (TN), total phosphorus (TP), and chlorophyll‐a (Chla). The samples were analyzed in the laboratory and involved the following: chlorophyll‐a (Chl‐a), using the 90% acetone extraction method. The determination of total phosphorus is conducted by the ammonium molybdate spectrophotometric method, the determination of total nitrogen by the alkaline potassium persulfate digestion‐ultraviolet spectrophotometric method, and the determination of ammonia nitrogen by the Nessler's reagent spectrophotometric method (GB3838‐2002 2002).

Phytoplankton and Rotifera were collected in 1‐l plastic bottles and then fixed with Lugol's iodine solution. The concentrated samples were precipitated and concentrated to 30 mL after 48 h. Samples used for assessing zooplankton (Copepoda, Cladocera) densities were collected by filtering a minimum of 10 L of water through a 64‐mm mesh from the different sampling points of the lake, and then concentrated into 50 mL plastic bottles and immediately preserved in the field in a 4% formaldehyde solution. We identified plankton species based on their morphological characteristics, and plankton individuals were classified at the lowest identifiable taxonomic level. Enumeration and identification were conducted in accordance with the microscopy method specified in methods for studying freshwater plankton (Zhang and Huang 1991), and each identified taxonomic name of the plankton was validated against the classification system proposed by Sun and Dongyan (1999) and (Wang et al. 1961; Jiang et al. 2000).

### 2.3 Data Analyses

We selected Shannon–Wiener's index, Pielou's evenness index, Margalef's index, and Simpson's diversity index to characterize the alpha diversity of phytoplankton and zooplankton. The differences in alpha diversity during various periods were calculated using the Mann–Whitney U test (*corrplot* package). To visualize compositional differences in plankton structure across periods, the Nonmetric Multidimensional Scaling (NMDS) ordination method based on the Bray–Curtis dissimilarity was used (*vegan* package). Similarity analysis based on zooplankton and phytoplankton abundance (ANOSIM) was performed to examine the differences in plankton communities between periods (*vegan* package). We analyzed the relationship between plankton community composition and environmental factors using redundancy analysis (RDA), ascertained the significance of the differences between the phytoplankton communities and the zooplankton communities and the environmental factors through a total of 999 permutations, and calculated the contribution of the environmental factors by hierarchical partitioning (*vegan* package; *rdacca. hp* package). Before the RDA analysis, we performed a Variance Inflation Factor analysis to eliminate the covariance, and a total of eight environmental factors were screened out: WT, Turb, DO, EC, WD, NH_3_, TP, Chla. In order to explore the influence of various factors on the distribution of plankton communities in different sampling periods, we performed Mantel test analysis by eliminating the covariance of environmental factors within the main dominant phytoplankton species (Chlorophyta, Cyanobacteria, Bacillariophyta, and Euglenophyta) and zooplankton (Rotifera, Copepoda, and Cladocera). In the process of the analysis, we introduced the concept of sampling time (time) (0 for pre‐heatwave period, 1 for heatwave period, 2 for heatwave taming period, 3 for post‐heatwave period), which was introduced to characterize the influence of heatwave together with water temperature (WT). To assess the effect of temperature on key species of the plankton community, we constructed a Linear Mixed Effects Model (LMM). The model identified environmental factors (DO, EC, Turb, SD, WD, NH_3_, TN, TP) and WT as fixed effects and sampling sites as random effects (*lme4* package; *glmm hp* package) (Lai et al. 2022, 2023). Prior to analysis, the plankton matrix and environmental factors matrix were downscaled using principal component analysis (PCA); the first principal component explained more than 50% of the overall variance, so we characterized the plankton community structure using PC1 and transformed water temperature by applying ln(*x* + 1) (*utils* package). When constructing the Linear Mixed Model (LMM), we verified the normality of residuals through Q‐Q plots to ensure the applicability of the model. All of the above analyses were performed in R (version 4.2.3) and their results were visualized using the *ggplot2* package.

Co‐occurrence networks are frequently used to identify interactions between community members. To construct a Co‐occurrence network analysis for viewing the interrelationships between plankton, we utilized CoNet (*igraph package*). Plankton taxa with a frequency of occurrence greater than 3 were selected for network analysis and a matrix was built based on Spearman's correlation coefficient. The topological properties of the CoNet were calculated using the *igraph package*. The definitions of relevant network attributes are as follows:

*Node*: Represents an individual phytoplankton/zooplankton species.

*Edge*: Represents the association between two nodes in the network, indicating the interaction between two species.

*Average degree*: The degree of a node counts the number of edges it has. The average degree is calculated over all nodes in the network.

*Clustering coefficient*: A parameter that reflects the close relationship among nodes in the network and embodies the cohesion of the network.

The results of the analysis were inspected using R (version 4.3.1) and visualized by Cytoscape (version 3.9.1).

We calculated within modular degree (Zi) and between modular degree (Pi) values to identify keystone species in the cluster. On the basis of Zi and Pi values, the nodes of each network were classified into different topological roles: module hubs (Zi ≥ 2.5, Pi < 0.62), network hubs (Zi ≥ 2.5, Pi ≥ 0.62), connectors (Zi < 2.5, Pi ≥ 0.62), and peripherals (Zi < 2.5, Pi < 0.62) (Meng et al. 2022) (*igraph* package). Apart from the peripherals, the other three categories are considered to be potentially key taxa as they play an important role in the network topology (Lukwambe et al. 2018). The analysis was performed employing R (version 4.3.1).

## 3 Results

### 3.1 Phytoplankton and Zooplankton Community Structure

**FIGURE 3:**
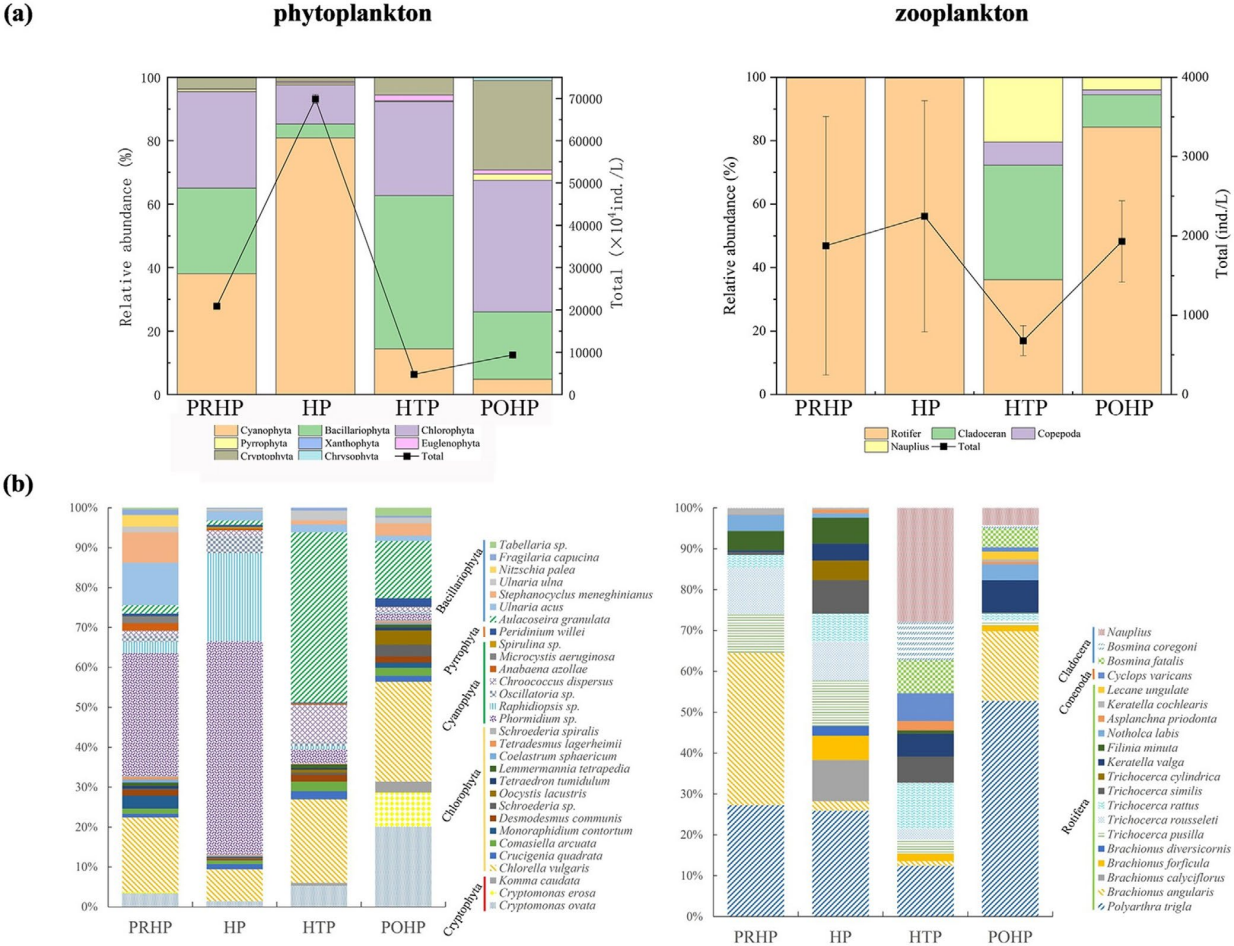
Variation in plankton taxa during each sampling period. (a) Variation in the relative abundance of different plankton taxa and total plankton density during sampling periods. The error bars represent the average of the standard deviations. (b) Bar chart showing the relative abundance of the top 30 species of phytoplankton, and the top 20 species of zooplankton. Abbreviations were used in the diagram: Pre‐heatwave period (PRHP), heatwave period (HP), heatwave taming period (HTP), and post‐heatwave period (POHP).

We identified a total of 152 phytoplankton and 53 zooplankton to species level from water samples collected from Shengjin Lake. From the pre‐heatwave period to the post‐heatwave period, the abundance of phytoplankton showed a trend of increasing first, then decreasing, and finally increasing again, with the lowest abundance of phytoplankton in the heatwave taming period. Chlorophyta, Cyanophyta, Bacillariophyta and Cryptophyta dominated the phytoplankton community in each period (Figure 3a). The abundance of phytoplankton increased in the heatwave period, and the abundance of Cyanophyta accounted for more than 80% of the total abundance, and then shifted to a Bacillariophyta‐dominated phytoplankton community in the heatwave taming period. Zooplankton abundance displayed a similar trend. The zooplankton community was dominated by Rotifera in the pre‐heatwave period and the heatwave period, and the abundance of large Copepoda and Cladocera increased in the heatwave taming period (Figure 3a).

In the analysis of the dominant species of plankton, we found that throughout all sampling periods, *Chlorella vulgaris, Ulnaria* sp., *and Polyarthra trigla* were always the most dominant species (Figure 3b). Regarding phytoplankton, during the pre‐heatwave period and the heatwave period, *Phormidium* sp. was the phytoplankton species with the highest proportion. However, during the heatwave taming period and the post‐heatwave period, the relative abundance of *Aulacoseira granulata* increased significantly, while the abundance of *Phormidium* sp. decreased. In terms of zooplankton, the relative abundances of *Cyclops varicans, Bosmina fatalis, Bosmina coregoni*, and *Nauplius* showed an upward trend during the heatwave taming period, but decreased during the post‐heatwave period. Overall, the changing trends of the main dominant species are consistent with the trends of the phytoplankton and zooplankton groups' abundance described above.

**FIGURE 4:**
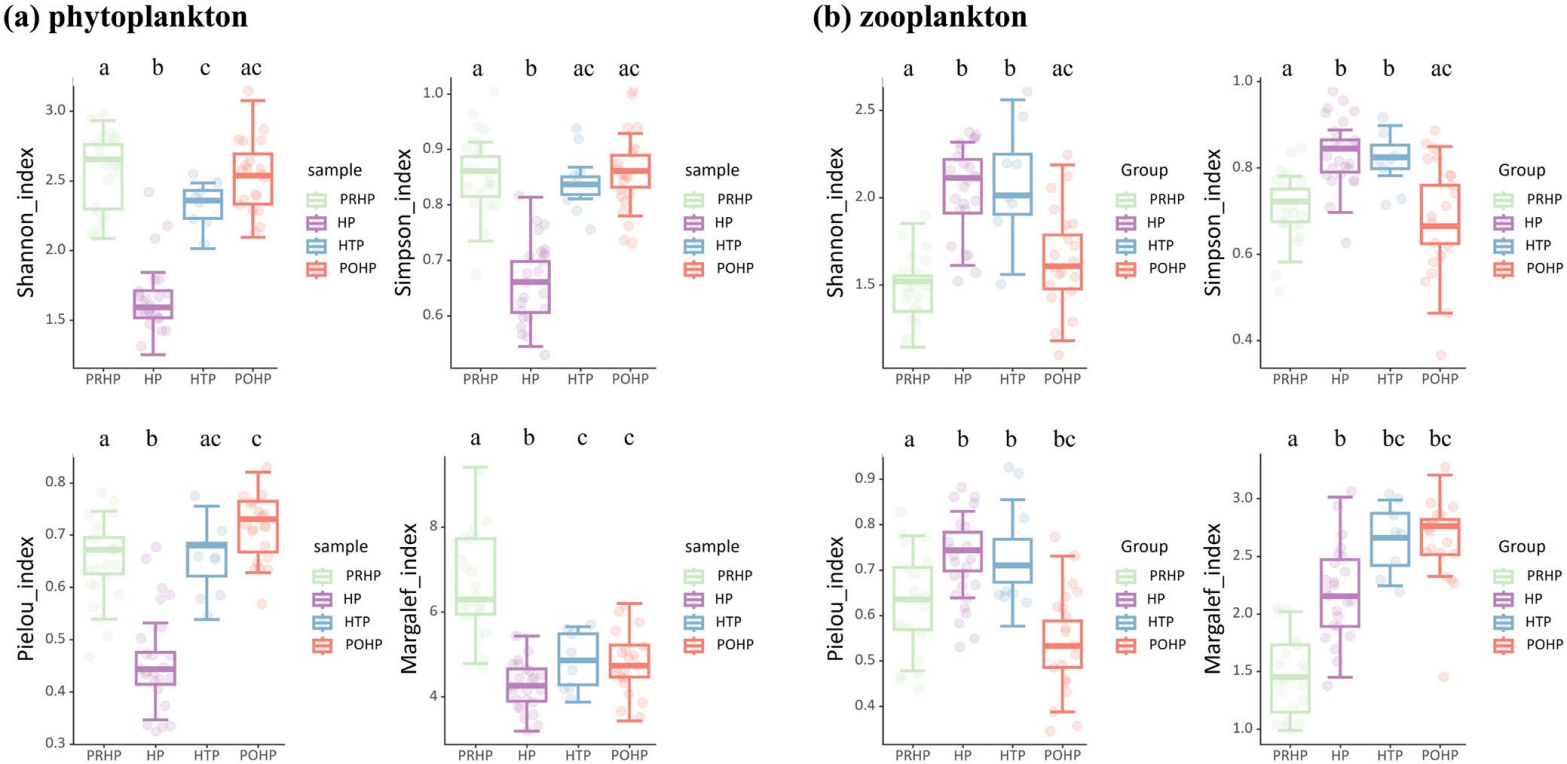
Box plot of the *α* diversity of plankton. Different lowercase letters indicate significant differences among sampling periods at a significance level of 0.05. The error bars represent the average of the standard deviations. Abbreviations were used in the diagram: Pre‐heatwave period (PRHP), heatwave period (HP), heatwave taming period (HTP), and post‐heatwave period (POHP).

The results of the study uncovered that the alpha diversity of phytoplankton and zooplankton varied significantly among sampling periods, with the diversity of the phytoplankton in the heatwave period being slightly lower than that in the other sampling periods, but it recovered to the previous level in the later periods (Figure 4).

**FIGURE 5:**
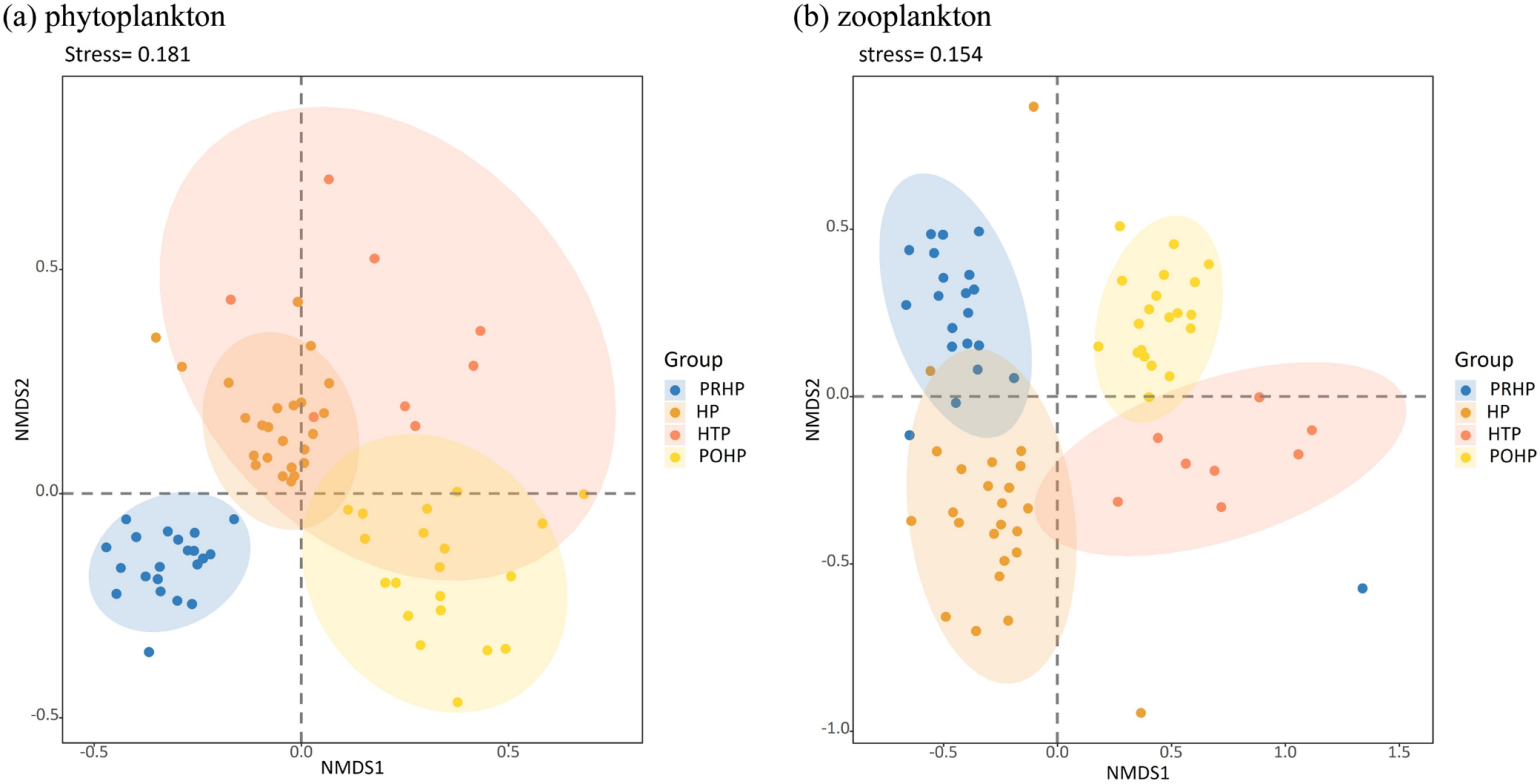
Nonmetric multidimensional scaling (NMDS) plots of the composition of phytoplankton and zooplankton during sampling periods based on Bray–Curtis similarities. Abbreviations were used in the diagram: Pre‐heatwave period (PRHP), heatwave period (HP), heatwave taming period (HTP), and post‐heatwave period (POHP).

In addition, we analyzed the compositional characteristics of phytoplankton and zooplankton at different sampling times. The NMDS results revealed a clear monthly aggregation of both phytoplankton and zooplankton (Figure 5). This was confirmed by ANOSIM (*r* = 0.7907, *p* < 0.001).

### 3.2 Driving Factors Influencing the Community Structure of Phytoplankton and Zooplankton

**FIGURE 6:**
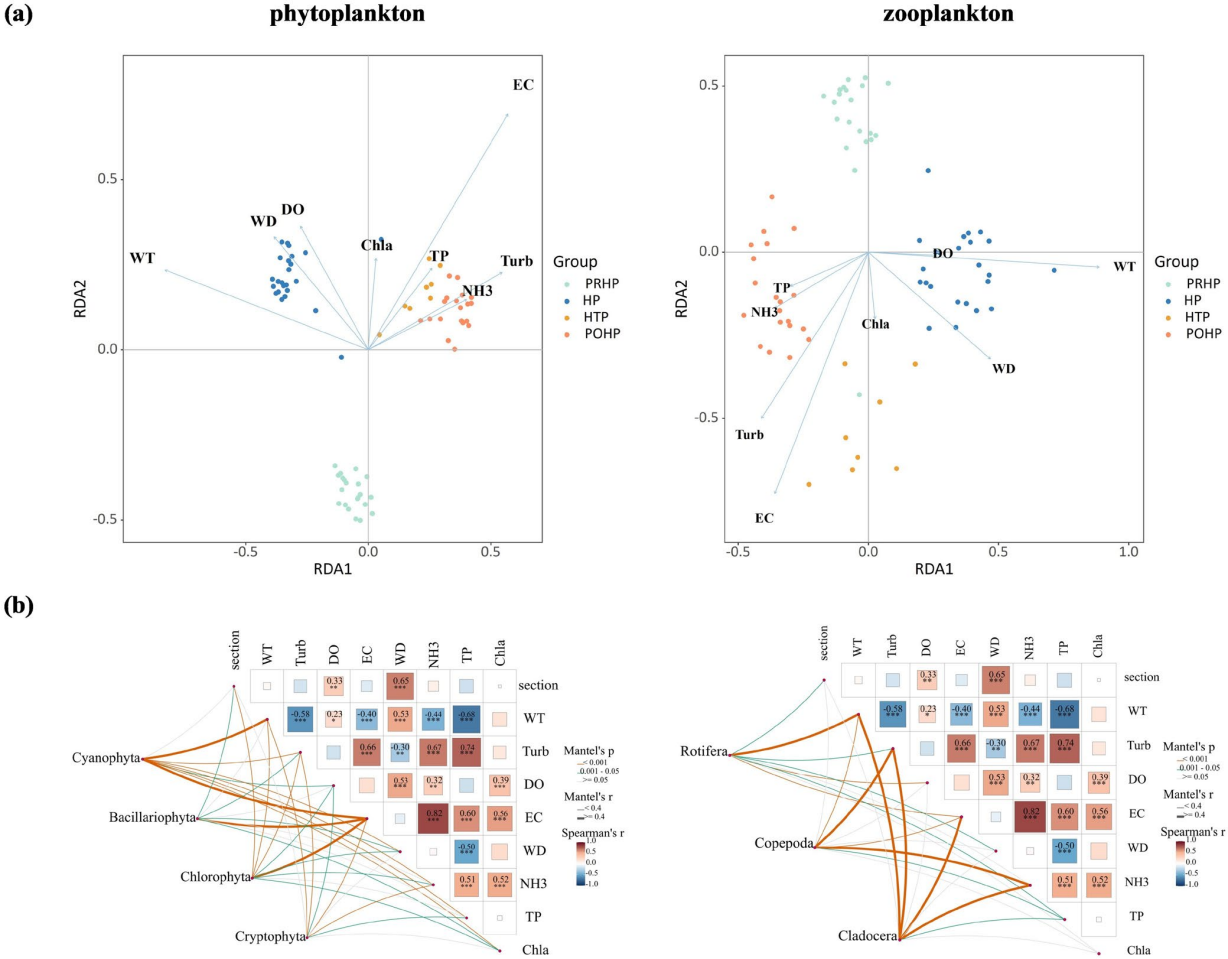
Relationship between environmental factors and plankton. (a) Bio‐plots of RDA results showing the relationships between phytoplankton zooplankton and environmental variables. (b) Heat map of correlation between the main dominant populations and environmental factors. Abbreviations were used in the diagram: Water temperature (WT), dissolved oxygen (DO), electrical conductivity (EC), turbidity (Turb), Secchi depth (SD), water depth (WD), ammonia (NH3), total nitrogen (TN), total phosphorus (TP), chlorophyll‐a (Chla), pre‐heatwave period (PRHP), heatwave period (HP), heatwave taming period (HTP), and post‐heatwave period (POHP).

**FIGURE 7:**
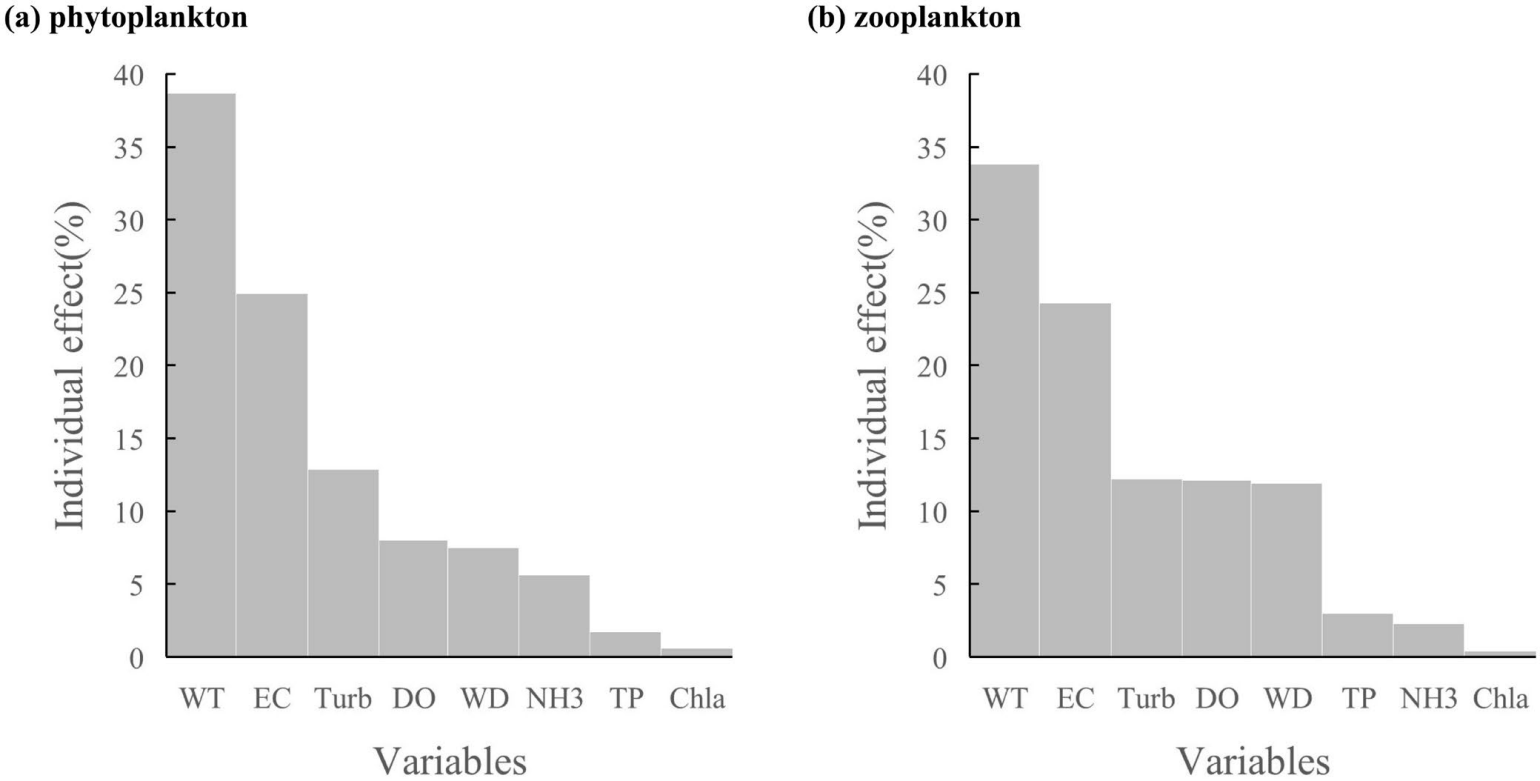
Bar chart of RDA hierarchical segmentation results. Abbreviations were used in the diagram: Water temperature (WT), dissolved oxygen (DO), electrical conductivity (EC), turbidity (Turb), Secchi depth (SD), water depth (WD), ammonia (NH3), total nitrogen (TN), total phosphorus (TP), chlorophyll‐a (Chla).

The results of redundancy analysis between zooplankton, phytoplankton and environmental factors showed that the sum of variance contribution rates of the first two axes was 71.06% and 85.01%, respectively (Figure 6). RDA analysis indicated that water temperature (WT), turbidity (Turb), electrical conductivity (EC), water depth (WD), and ammonia nitrogen (NH_3_) were the core environmental factors affecting zooplankton and phytoplankton communities (*p* < 0.001), while total phosphorus (TP) was highly significantly correlated with zooplankton and phytoplankton communities (*p* < 0.01). Furthermore, hierarchical partitioning confirmed that water temperature was the most critical driving factor, contributing 33.83% and 38.70% to the changes in zooplankton and phytoplankton communities, respectively (Figure 7).

In order to determine the key factors influencing the plankton community during different sampling periods, we performed Mantel test analysis by eliminating the covariance of environmental factors within the main dominant phytoplankton species (Chlorophyta, Cyanobacteria, Bacillariophyta, and Euglenophyta) and zooplankton (Rotifera, Copepoda, and Cladocera) (Table 1). The results uncovered that sampling time had a highly significant correlation for Chlorophyta, Cyanobacteria, and Bacillariophyta and water temperature impacted Chlorophyta. In addition, the effect of sampling time on Copepoda and Cladocera community structure was also pronounced, and water temperature was highly correlated with Rotifera and Cladocera (Figure 6b). The data of environmental factors during the sampling periods are shown in Table 1.

**TABLE 1:** Environmental factors characteristics of the four periods (mean value ± SD).

Parameters	PRHP	HP	HTP	POHP
Water temperature (°C)	22.57 ± 0.72	30.14 ± 0.59	34.35 ± 0.85	27.94 ± 0.90
Water depth (m)	1.90 ± 0.56	2.11 ± 0.48	2.61 ± 0.66	1.80 ± 0.51
Secchi depth (cm)	29.45 ± 5.76	22.50 ± 17.18	76.92 ± 22.41	52.19 ± 10.10
pH	7.7 ± 0.11	7.71 ± 0.33	8.81 ± 0.43	8.47 ± 0.17
Turbidity (NTU)	44.68 ± 15.72	85.44 ± 52.07	8.68 ± 4.39	10.26 ± 6.07
Electrical conductivity (μs/cm)	242.15 ± 8.41	217.71 ± 8.68	184.64 ± 7.98	129.85 ± 4.28
Dissolved oxygen (mg/L)	9.43 ± 0.96	6.25 ± 1.18	10.10 ± 1.77	7.98 ± 0.24
Total nitrogen (mg/L)	1.62 ± 0.51	0.08 ± 0.02	0.17 ± 0.02	0.08 ± 0.01
Ammonia (mg/L)	0.95 ± 1.24	0.04 ± 0.01	0.04 ± 0.03	0.02 ± 0.01
Total phosphorus (mg/L)	0.12 ± 0.03	0.07 ± 0.02	0.06 ± 0.13	0.03 ± 0.01
Chla (μg/L)	13.35 ± 24.16	8.60 ± 6.37	12.28 ± 22.19	1.92 ± 1.52

Abbreviations: HP, heatwave period; HTP, heatwave taming period; POHP, post‐heatwave period; PRHP, Pre‐heatwave period.

### 3.3 Interactions Between Phytoplankton and Zooplankton Communities

**FIGURE 8:**
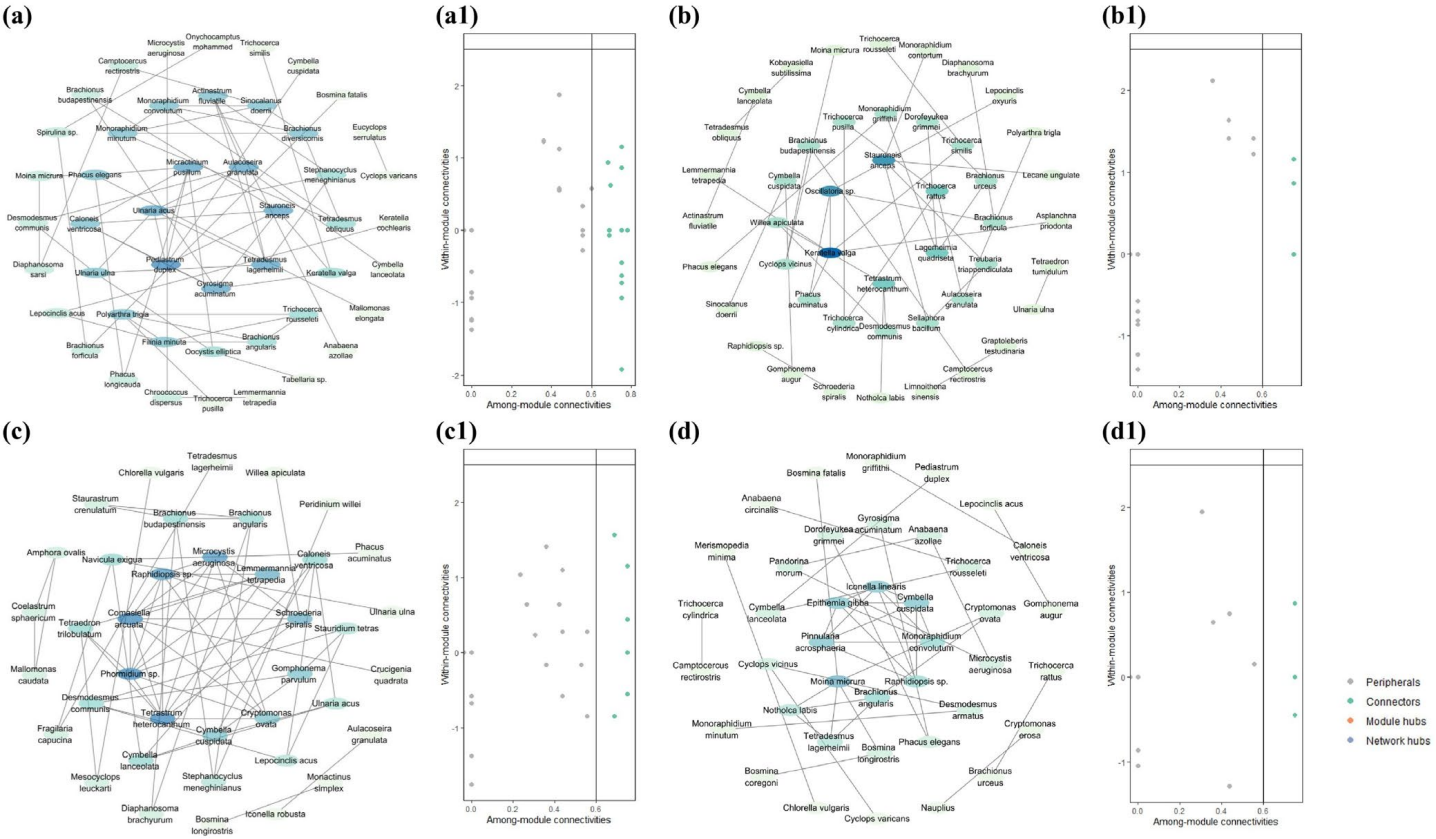
Co‐occurrence network information of the plankton community in different sampling periods. (a–d) The co‐occurrence network structure of plankton community at different sampling periods. Circles represent network nodes, and connecting lines represent the connections between nodes. The green circles range from dark to light in color, corresponding to a decrease in degree values from high to low. (a1–d1) Zi‐Pi plots indicated the distribution characteristics of species based on their topological roles in four networks. The threshold values of Zi and Pi for categorizing species were 2.5 and 0.62, respectively. Abbreviations were used in the diagram: Pre‐heatwave period (PRHP), heatwave period (HP), heatwave taming period (HTP), and post‐heatwave period (POHP).

We generated interaction networks between phytoplankton and zooplankton to analyze the variability between interrelationships between zooplankton in different sampling periods (Figure 8a–d). The results revealed that as the heatwave progressed, the network properties in the four sampling periods underwent changes. (Table 2). There were fewer network nodes in the heatwave taming period than in the other three sampling times, and significantly fewer zooplankton network nodes. The clustering coefficient and average degree were higher than in the pre‐heatwave period and the heatwave period. This indicates that the phytoplankton–zooplankton network connectivity was higher and more aggregated in the heatwave taming period. Average degree in the post‐heatwave period was not as high as the heatwave taming period's, but the clustering coefficient was greater, signaling that the network connectivity in the post‐heatwave period was not as high as the network connectivity in the heatwave taming period, but the communities were more tightly knit.

**TABLE 2:** Co‐network characteristics of phytoplankton and zooplankton.

	PRHP	HP	HTP	POHP
Network node	50	46	39	40
Zooplankton	19	20	5	14
Phytoplankton	31	26	34	26
Edge	66	39	65	43
Positive	65	38	65	38
Negative	1	1	0	5
Positive link percentage (%)	98.48	97.44	100	88.37
Average degree	2.64	1.70	3.33	2.15
Clustering coefficient	0.44	0.32	0.69	0.76

Abbreviations: HP, heatwave period; HTP, heatwave taming period; POHP, post‐heatwave period; PRHP, Pre‐heatwave period.

In our study, more than 95% of the zooplankton network links in the first three sampling periods were positively linked, suggesting that zooplankton were mutually reinforcing during this period (Table 2). In the post‐heatwave period, the proportion of positive links in the zooplankton mutualism network decreased to 88.37%, and negative links emerged during this period.

We further dissected the composition of the phytoplankton–zooplankton interactions network in the planktonic ecosystem (Figure 8a–d, Table 2, Table 3). Bacillariophyta and Chlorophyta dominated the phytoplankton nodes in the heatwave taming period. In the zooplankton nodes, Rotifera proliferated in the pre‐heatwave period and the heatwave period (57.89% and 65%). However, by the heatwave taming period and the post‐heatwave period, Copepoda and Cladocera increased in their share of zooplankton network nodes (60% and 57.14%). Information regarding the network nodes of plankton can be found in Table 3.

**TABLE 3:** Information regarding the network nodes of plankton.

PRHP			HP			HTP			POHP		
node_ID	Phylum/Class	Topological role	node_ID	Phylum/Class	Topological role	node_ID	Phylum/Class	Topological role	node_ID	Phylum/Class	Topological role
*Brachionus forficula*	Rotifera	Connector	*Brachionus urceus*	Rotifera	Connector	*Brachionus angularis*	Rotifera	Peripherals	*Brachionus urceus*	Rotifera	Peripherals
*Brachionus angularis*	Rotifera	Peripherals	*Brachionus forficula*	Rotifera	Connector	*Brachionus budapestinensis*	Rotifera	Peripherals	*Brachionus angularis*	Rotifera	Peripherals
*Brachionus budapestinensis*	Rotifera	Connector	*Brachionus budapestinensis*	Rotifera	Connector	*Mesocyclops leuckarti*	Copepoda	Connector	*Notholca labis*	Rotifera	Peripherals
*Keratella cochlearis*	Rotifera	Peripherals	*Keratella valga*	Rotifera	Peripherals	*Diaphanosoma brachyurum*	Cladocera	Connector	*Trichocerca rousseleti*	Rotifera	Connector
*Keratella valga*	Rotifera	Peripherals	*Notholca labis*	Rotifera	Peripherals	*Bosmina longirostris*	Cladocera	Peripherals	*Trichocerca rattus*	Rotifera	Peripherals
*Brachionus diversicornis*	Rotifera	Peripherals	*Asplanchna priodonta*	Rotifera	Peripherals	*Microcystis aeruginosa*	Cyanophyta	Peripherals	*Trichocerca cylindrica*	Rotifera	Peripherals
*Trichocerca similis*	Rotifera	Peripherals	*Lecane ungulate*	Rotifera	Peripherals	*Phormidium* sp.	Cyanophyta	Peripherals	*Cyclops vicinus*	Copepoda	Connector
*Trichocerca rousseleti*	Rotifera	Peripherals	*Trichocerca similis*	Rotifera	Connector	*Raphidiopsis* sp.	Cyanophyta	Peripherals	*Cyclops varicans*	Copepoda	Peripherals
*Trichocerca pusilla*	Rotifera	Peripherals	*Trichocerca rousseleti*	Rotifera	Peripherals	*Fragilaria capucina*	Bacillariophyta	Connector	*Nauplius*	Copepoda	Peripherals
*Polyarthra trigla*	Rotifera	Peripherals	*Trichocerca pusilla*	Rotifera	Connector	*Cymbella lanceolata*	Bacillariophyta	Connector	*Moina micrura*	Cladocera	Peripherals
*Filinia minuta*	Rotifera	Peripherals	*Trichocerca rattus*	Rotifera	Peripherals	*Cymbella cuspidata*	Bacillariophyta	Peripherals	*Camptocercus rectirostris*	Cladocera	Peripherals
*Cyclops varicans*	Copepoda	Peripherals	*Trichocerca cylindrica*	Rotifera	Connector	*Iconella robusta*	Bacillariophyta	Peripherals	*Bosmina fatalis*	Cladocera	Peripherals
*Eucyclops serrulatus*	Copepoda	Peripherals	*Polyarthra trigla*	Rotifera	Peripherals	*Amphora ovalis*	Bacillariophyta	Connector	*Bosmina coregoni*	Cladocera	Peripherals
*Onychocamptus mohammed*	Copepoda	Peripherals	*Cyclops vicinus*	Copepoda	Connector	*Stephanocyclus meneghinianus*	Bacillariophyta	Peripherals	*Bosmina longirostris*	Cladocera	Connector
*Sinocalanus doerrii*	Copepoda	Peripherals	*Sinocalanus doerrii*	Copepoda	Peripherals	*Gomphonema parvulum*	Bacillariophyta	Peripherals	*Merismopedia minima*	Cyanophyta	Peripherals
*Moina micrura*	Cladocera	Connector	*Limnoithona sinensis*	Copepoda	Peripherals	*Caloneis ventricosa*	Bacillariophyta	Peripherals	*Microcystis aeruginosa*	Cyanophyta	Connector
*Camptocercus rectirostris*	Cladocera	Connector	*Moina micrura*	Cladocera	Peripherals	*Ulnaria acus*	Bacillariophyta	Peripherals	*Raphidiopsis* sp.	Cyanophyta	Peripherals
*Diaphanosoma sarsi*	Cladocera	Connector	*Graptoleberis testudinaria*	Cladocera	Peripherals	*Ulnaria ulna*	Bacillariophyta	Peripherals	*Anabaena azollae*	Cyanophyta	Connector
*Bosmina fatalis*	Cladocera	Peripherals	*Camptocercus rectirostris*	Cladocera	Peripherals	*Aulacoseira granulata*	Bacillariophyta	Peripherals	*Anabaena circinalis*	Cyanophyta	Peripherals
*Spirulina* sp.	Cyanophyta	Connector	*Diaphanosoma brachyurum*	Cladocera	Peripherals	*Navicula exigua*	Bacillariophyta	Connector	*Gyrosigma acuminatum*	Bacillariophyta	Connector
*Chroococcus dispersus*	Cyanophyta	Connector	*Oscillatoria* sp.	Cyanophyta	Peripherals	*Schroederia spiralis*	Chlorophyta	Peripherals	*Cymbella lanceolata*	Bacillariophyta	Connector
*Microcystis aeruginosa*	Cyanophyta	Peripherals	*Raphidiopsis* sp.	Cyanophyta	Peripherals	*Staurastrum crenulatum*	Chlorophyta	Connector	*Cymbella cuspidata*	Bacillariophyta	Peripherals
*Anabaena azollae*	Cyanophyta	Peripherals	*Stauroneis anceps*	Bacillariophyta	Peripherals	*Coelastrum sphaericum*	Chlorophyta	Peripherals	*Iconella linearis*	Bacillariophyta	Peripherals
*Gyrosigma acuminatum*	Bacillariophyta	Peripherals	*Cymbella lanceolata*	Bacillariophyta	Peripherals	*Stauridium tetras*	Chlorophyta	Peripherals	*Gomphonema augur*	Bacillariophyta	Peripherals
*Stauroneis anceps*	Bacillariophyta	Peripherals	*Cymbella cuspidata*	Bacillariophyta	Peripherals	*Monactinus simplex*	Chlorophyta	Peripherals	*Epithemia gibba*	Bacillariophyta	Peripherals
*Tabellaria* sp.	Bacillariophyta	Peripherals	*Gomphonema augur*	Bacillariophyta	Peripherals	*Crucigenia quadrata*	Chlorophyta	Peripherals	*Pinnularia acrosphaeria*	Bacillariophyta	Peripherals
*Cymbella lanceolata*	Bacillariophyta	Peripherals	*Ulnaria ulna*	Bacillariophyta	Peripherals	*Willea apiculata*	Chlorophyta	Peripherals	*Caloneis ventricosa*	Bacillariophyta	Peripherals
*Cymbella cuspidata*	Bacillariophyta	Peripherals	*Aulacoseira granulata*	Bacillariophyta	Connector	*Lemmermannia tetrapedia*	Chlorophyta	Peripherals	*Dorofeyukea grimmei*	Bacillariophyta	Peripherals
*Stephanocyclus meneghinianus*	Bacillariophyta	Peripherals	*Dorofeyukea grimmei*	Bacillariophyta	Peripherals	*Tetraedron trilobulatum*	Chlorophyta	Peripherals	*Monoraphidium griffithii*	Chlorophyta	Peripherals
*Caloneis ventricosa*	Bacillariophyta	Peripherals	*Sellaphora bacillum*	Bacillariophyta	Peripherals	*Tetrastrum heterocanthum*	Chlorophyta	Peripherals	*Pediastrum duplex*	Chlorophyta	Peripherals
*Ulnaria acus*	Bacillariophyta	Peripherals	*Kobayasiella subtilissima*	Bacillariophyta	Peripherals	*Chlorella vulgaris*	Chlorophyta	Peripherals	*Pandorina morum*	Chlorophyta	Connector
*Ulnaria ulna*	Bacillariophyta	Connector	*Monoraphidium griffithii*	Chlorophyta	Peripherals	*Tetradesmus lagerheimii*	Chlorophyta	Peripherals	*Monoraphidium convolutum*	Chlorophyta	Peripherals
*Aulacoseira granulata*	Bacillariophyta	Peripherals	*Lagerheimia quadriseta*	Chlorophyta	Peripherals	*Comasiella arcuata*	Chlorophyta	Peripherals	*Chlorella vulgaris*	Chlorophyta	Peripherals
*Schroederia spiralis*	Chlorophyta	Connector	*Schroederia spiralis*	Chlorophyta	Peripherals	*Desmodesmus communis*	Chlorophyta	Peripherals	*Monoraphidium minutum*	Chlorophyta	Peripherals
*Actinastrum fluviatile*	Chlorophyta	Peripherals	*Actinastrum fluviatile*	Chlorophyta	Peripherals	*Peridinium willei*	Pyrrophyta	Peripherals	*Tetradesmus lagerheimii*	Chlorophyta	Peripherals
*Oocystis elliptica*	Chlorophyta	Connector	*Willea apiculata*	Chlorophyta	Peripherals	*Phacus acuminatus*	Euglenophyta	Peripherals	*Desmodesmus armatus*	Chlorophyta	Peripherals
*Pediastrum duplex*	Chlorophyta	Connector	*Lemmermannia tetrapedia*	Chlorophyta	Peripherals	*Lepocinclis acus*	Euglenophyta	Peripherals	*Phacus elegans*	Euglenophyta	Connector
*Lemmermannia tetrapedia*	Chlorophyta	Peripherals	*Treubaria triappendiculata*	Chlorophyta	Peripherals	*Cryptomonas ovata*	Cryptophyta	Peripherals	*Lepocinclis acus*	Euglenophyta	Peripherals
*Micractinium pusillum*	Chlorophyta	Peripherals	*Tetraedron tumidulum*	Chlorophyta	Peripherals	*Mallomonas caudata*	Chrysophyta	Peripherals	*Cryptomonas ovata*	Cryptophyta	Peripherals
*Monoraphidium convolutum*	Chlorophyta	Peripherals	*Tetrastrum heterocanthum*	Chlorophyta	Peripherals				*Cryptomonas erosa*	Cryptophyta	Peripherals
*Monoraphidium minutum*	Chlorophyta	Peripherals	*Monoraphidium contortum*	Chlorophyta	Peripherals						
*Tetradesmus lagerheimii*	Chlorophyta	Peripherals	*Tetradesmus obliquus*	Chlorophyta	Peripherals						
*Tetradesmus obliquus*	Chlorophyta	Peripherals	*Desmodesmus communis*	Chlorophyta	Peripherals						
*Desmodesmus communis*	Chlorophyta	Peripherals	*Phacus elegans*	Euglenophyta	Peripherals						
*Peridinium willei*	Pyrrophyta	Peripherals	*Phacus acuminatus*	Euglenophyta	Connector						
*Ophiocytium capitatum*	Xanthophyta	Peripherals	*Lepocinclis oxyuris*	Euglenophyta	Peripherals						
*Phacus elegans*	Euglenophyta	Peripherals									
*Phacus longicauda*	Euglenophyta	Connector									
*Lepocinclis acus*	Euglenophyta	Peripherals									
*Mallomonas elongata*	Chrysophyta	Peripherals									

Abbreviations: HP, heatwave period; HTP, heatwave taming period; POHP, post‐heatwave period; PRHP, Pre‐heatwave period.

### 3.4 Water Temperature Shapes Keystone Species in the Phytoplankton–Zooplankton Interaction Network

Connectors, modular hubs and network hubs were identified as keystone species in the community. In our study, the keystone species is the presence of connectors, while modular hubs and network hubs did not appear in our networks. We found 16 connectors in the plankton community in the pre‐heatwave period, 16 connectors in the heatwave period, 9 connectors in the heatwave taming period, and 12 connectors in PREP (Figure 8a1–d1, Table 3). The results indicate significantly lower numbers of keystone species in the heatwave taming period than in the other three periods. During our sampling periods, the phytoplankton keystone species shifted from predominantly Chlorophyta to Bacillariophyta, and the zooplankton keystone species shifted from Rotifera dominated to Cladocera and Copepoda. These results revealed that 33.3% and 36.4% of the phytoplankton keystone species (connectors) belonged to Bacillariophyta and Chlorophyta, respectively, and that Rotifera comprised 50% of the zooplankton keystone species.

**FIGURE 9:**
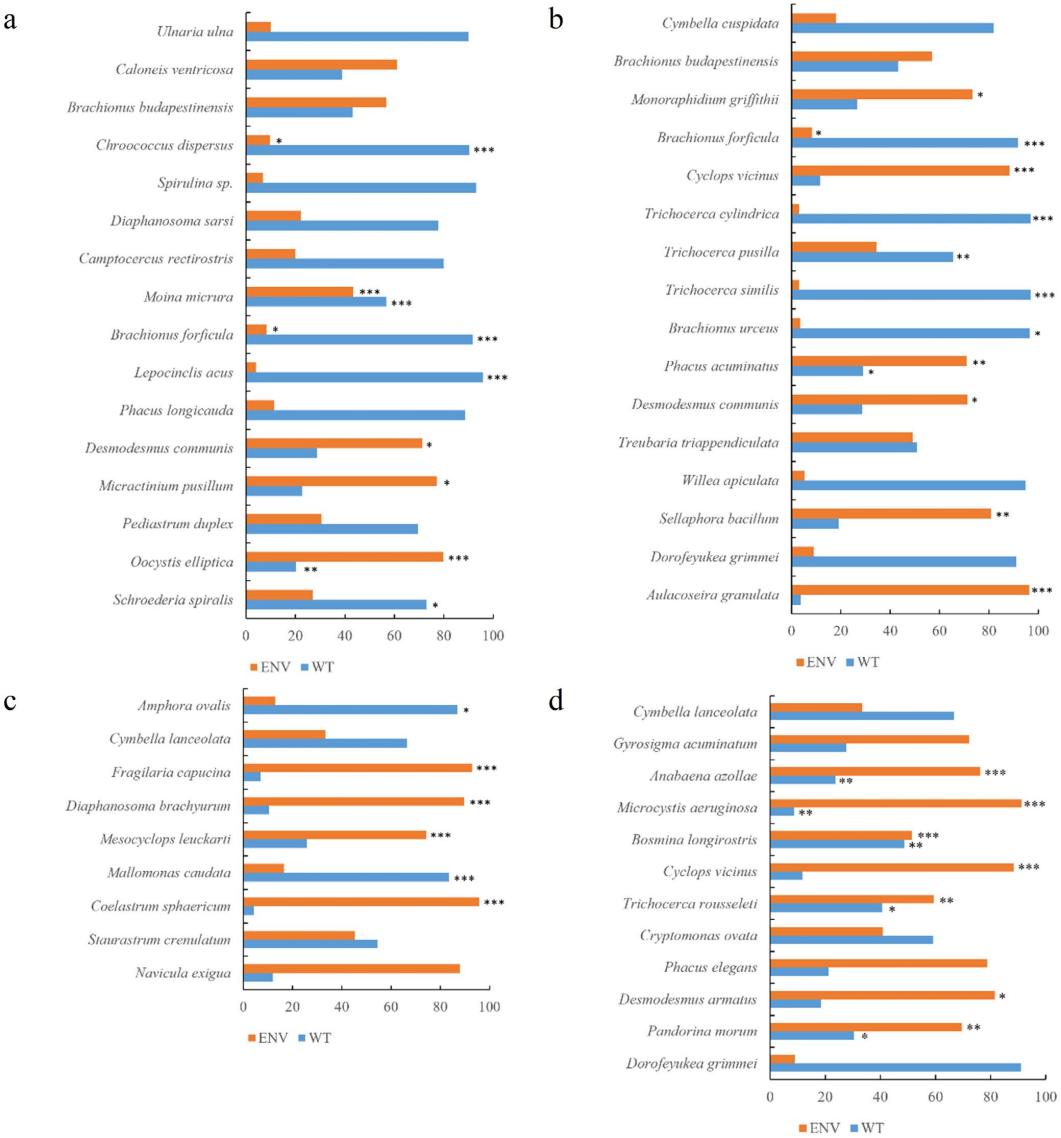
Effects of water temperature and environmental factors on key species can range from 1% to 100% in the abscissa. Significance is represented by asterisks. (***p < 0.001, **p < 0.01, *p < 0.05). Abbreviations were used in the diagram: Pre‐heatwave period (PRHP), heatwave period (HP), heatwave taming period (HTP), and post‐heatwave period (POHP).

To further explore the effects of environmental factors on the phytoplankton–zooplankton dichotomous network, we used an LMM to test the effects of water temperature and environmental variables on the key species of the network (Figure 9). The results indicated that during the pre‐heatwave period and the heatwave period, the proportion of keystone species that responded significantly to water temperature exceeded 50%, with the respective proportions reaching 68.8% and 56.3%. Notably, after entering the heatwave taming period, this proportion decreased markedly to 44.4%; and by the post‐heatwave period, the proportion of keystone species showing significant responses to water temperature dropped further to 25%.

## 4 Discussion

### 4.1 Impacts of Sustained Heatwaves on Zooplankton and Phytoplankton Community Structure

Cyanophyta were of high relative abundance in the heatwave period, which is consistent with previous studies that showed that lakes in the middle and lower reaches of the Yangtze River experience blooms at high temperatures in summer (Rao et al. 2021; Su et al. 2019). In most studies, Bacillariophyta emerges as the dominant taxon in the region in fall and winter, with a decrease in relative abundance in spring and summer (Fu and Sun 2024; Hou et al. 2025; Lv et al. 2023; Wang et al. 2009). However, in our study, the relative abundance of Bacillariophyta increased in the heatwave taming period (the hottest period within the study area), replacing the dominance of the Cyanophyta, which is contrary to the above studies. The reason for this phenomenon is due to the high abundance of thermophilic species of *Aulacoseira granulata* and its variants. The results of this study were confirmed by the related study, in which the increase in water temperature in summer decreased the relative abundance of the unicellular eutrophic center diatoms *Acanthoceras* sp., *Asterionella* sp., and increased the filamentous diatom *Aulacoseira* sp. and *Tribonema* sp. in the phytoplankton (Abonyi et al. 2018). In the general environmental context of global warming, *Aulacoseira* sp. is characterized by a higher tolerance to high temperatures (Jea et al. 2017; Li et al. 2021). In addition, moderate water level fluctuations were favorable for algal blooms (Li et al. 2015). In August, in East China, precipitation decreased under the influence of La Niña, and rainfall washed away fewer phytoplankton, which was conducive to the growth and reproduction of *Aulacoseira granulata* as a dominant species (Zou et al. 2018). The water level of Lake Shengjin was affected by high temperatures and desertification, which made the water body lower than in previous years, and the water body was susceptible to wind disturbance, which decreased the deposition of *Aulacoseira granulata* , and thus, maintained high abundance of *Aulacoseira granulata* in the water column in the heatwave taming period.

In the context of global warming, the gradual dominance of small organisms and the progressive miniaturization of organisms have been noted in many reports (Álvarez and Ruano 2024; Bazin et al. 2023). This pattern extends from the individual to the community level, and the same applies to the zooplankton community, where high temperatures can drive the size distribution of the zooplankton community towards smaller individuals with more rapid turnover and lower biomass. That is, the larger‐bodied Cladocera and Copepoda communities will be replaced by smaller‐bodied Rotifera communities in a high‐temperature environment, which means that Rotifera have higher heat tolerance (Sommer et al. 2017; Uszko et al. 2022). In our study, Rotifera emerged as the predominant group among the dominant species during the heatwave period. Furthermore, in addition to having higher heat tolerance, Rotifera may benefit from reduced predation pressure and competitive release due to lower abundance of Cladocera and Copepoda (Sanna et al. 2013; Strecker and Vinebrooke 2004). However, all of these studies only address a range of effects of high temperatures and do not consider the effects of a persistent heatwave on the zooplankton community. In this study, the zooplankton community in the heatwave taming period under high‐temperature stress showed the exact opposite of the above phenomenon: in August 2022 (the heatwave taming period), Lake Shengjin was still under heatwave stress, but the relative abundance of large zooplankton (Cladocera and Copepoda) increased, the abundance and biomass of Cladocera and Copepoda increased, and the dominant species gradually appeared as Cladocera and Copepoda. Due to the short generation cycle of zooplankton, prolonged high‐temperature duration events can affect zooplankton life‐history strategies, which is different from the results brought by high temperature alone (Burton et al. 2020; Hermann et al. 2024). In a laboratory setting, Cladocera is heat domesticated to prolonged high‐temperature shock and has heat‐tolerant phenotypic plasticity (Van Baelen et al. 2024). In the context of sustained high temperatures in the heatwave period and the heatwave taming period at Lake Shengjin, large zooplankton reached laboratory‐like thermally selective conditions in their natural environment after a sustained high‐temperature heatwave lasting more than 50 days. We speculate that this process might enhance the high‐temperature tolerance of large zooplankton (Cladocera and Copepoda).

Dormancy in seasonal climates is a common strategy for building the diversity of organisms. Climate change may alter the dormancy dynamics of zooplankton, with high temperatures and long photoperiods promoting the hatching of dormant eggs of large zooplankton (Gyllström and Hansson 2004) a pattern that is validated by the number of nauplius in the heatwave taming period. Another possible reason for the expansion in the number of nauplius is that under heatwave stress, Cladocera and Copepoda reach maturity faster and reproduce as much as possible in response to the harsh environment (Daufresne et al. 2009).

The phenomenon of the persistent heatwave ended in September (post‐heatwave period), but the phytoplankton community responded to the environment with a certain lag (De Frenne et al. 2013), so the community structure was still characterized by the most dominant species of *Aulacoseira granulata* of Bacillariophyta. In addition, as the temperature receded, the unicellular algae in the phytoplankton community, such as *Chlorella vulgaris, Stephanocyclus meneghinianus*, and *Cryptomonas* sp., reached appropriate temperature and reproduced in large quantities, which promoted the development of the Rotifera community that preferred to feed on them, and the relative abundance of Rotifera increased (Hansson et al. 2007).

### 4.2 Water Temperature Shapes Keystones in the Plankton Network

From a topological point of view, different species play different roles in the networks under study. Some species act as connectors, so they can function as keystone species and influence community composition and maintenance of ecological functions (Banerjee et al. 2018). Our study discovered that the proportion of keystone species that responded dramatically to temperature during the heatwave taming period was gradually decreasing with the development of a sustained heatwave. More notably, *Diaphanosoma brachyurum, Mesocyclops leuckarti, and Coelastrum sphaericum*, which act as keystone species in the heatwave taming period, have wider ecological niche widths and were found in different aquatic periods. This can be mainly attributed to two points: First, high temperatures are antagonistic for most plankton and inhibit plankton growth and development (Sun and Arnott 2022). Second, species that can survive in harsh habitats have a wider habitat breadth and are insensitive to environmental changes (Chen et al. 2022; Jordán 2009). In other words, taxa with narrower habitat ecotope widths may face strong negative environmental selection and be unable to tolerate the hazards of high‐temperature stress. During community succession, species with narrow thermal niches may decline as environmental conditions fluctuate, allowing generalist species with broader niche widths to persist or dominate.

In addition, we also found that although the α diversity of zooplankton and phytoplankton decreased during the heatwave period, it recovered to the previous level afterward. Plankton communities typically have high population densities and individuals with multifunctional physiology contribute to resistance to diversity loss (D'Alelio et al. 2016). In biological communities, keystone species that are highly connected and play an important role in community structure and function can increase the functional redundancy of the community and further increase the biological buffering capacity to resist the impacts of heatwave events on aquatic ecosystems (Beynon et al. 2012; Mori et al. 2017). There are immigration and emigration of keystone species as well as the recovery of dormant species in plankton communities. Even if some sensitive keystone species are affected during the heatwave period, the community can still gradually restore functional redundancy through species turnover (e.g., the immigration of heat‐tolerant keystone species and the recovery of dormant species to fill ecological niches), and ultimately maintain overall diversity and the stability of the ecological network (Reise et al. 2006). Although extreme events such as heatwaves may lead to changes in species composition, the level of ecosystem functioning (e.g., productivity and energy flow) may be restored through this functional redundancy (Peralta et al. 2014). This means that ecosystems are able to maintain their basic functions even when species composition changes. Therefore, composition changed and diversity declined during the heatwave, but diversity recovered by the end of the study.

High temperature stress may have devastating effects on species in aquatic ecosystems and may even lead to the extinction of the species in that community (Ainsworth et al. 2020). Our study shows a transient decline in plankton alpha diversity and the number of network nodes during the heatwave period. In the face of extreme climatic events, food webs within ecosystems may undergo reconnection, which may involve the loss of species, the introduction of new species, or changes in species interactions, after which ecosystem diversity may return to its original level, as suggested by Folke (2006). The mechanisms underpinning the potential recovery deserve further investigation.

### 4.3 Impacts of Sustained Heatwaves on Phytoplankton–Zooplankton Interactions in Aquatic Networks

The phytoplankton–zooplankton interactions network relationship is determined by the simultaneous top‐down and bottom‐up effects (Robinson et al. 2024). In this study, the plankton interactions network links, except for September, were mostly positively correlated with each other, but the proportion of negative links increased during the later part of the heatwave, suggesting a rise in predation pressure in the community, which may have been enhanced by the top‐down action of zooplankton (Anderson and Harvey 2019; Sherr and Sherr 2007). Warmer water temperatures streamline the structure of freshwater ecosystem food webs (O'Gorman et al. 2019). It has been shown that warming increases consumer metabolism and shortens energy flow paths between consumers and producers, which in turn reduces the complexity of the network structure (Zhao et al. 2023). Our results are consistent with those of the above‐mentioned scholars. The average degree and clustering coefficient during the heatwave period were the lowest among the survey periods, indicating that extreme events in ecosystems lead to a reduction in the complexity of ecosystem networks.

For zooplankton, the growth of phytoplankton will change the predation pressure and thus indirectly affect the distribution of the zooplankton community structure (de Araújo et al. 2024; Robinson et al. 2024). In the pre‐heatwave period and the heatwave period, the main phytoplankton groups were dominated by Cyanophyta and Chlorophyta, among which were Cyanophyta species of the genus *Phormidium*. Some filamentous Cyanophyta (e.g., the genus *Phormidium*) can clog the filter‐feeding apparatus of large zooplankton and secrete phycotoxins; such toxins are toxic to Cladocera and Copepoda, thus inhibiting their development (Jef et al. 2018; Nagel et al. 2016; Samba et al. 2012). The absence of competitors promoted the development of Rotifera (during the pre‐heatwave period and heatwave period, both the abundance and nodes of zooplankton were dominated by Rotifera).

Sustained heatwaves can alter the structure of predation in the plankton community. The dominant species of Rotifera and Cladocera in our survey area were mainly filter feeders, feeding on algae, bacteria, and organic detritus in the water column, while the main species of Copepoda were dominated by the carnivorous species *Cyclops* sp. As the heat wave subsides, the water temperature, though still relatively high, has gradually stabilized. The earlier heat acclimation has endowed the dominant species of Cladocera with more favorable conditions for development, leading to an increase in their population. The growing number of Cladocera provides a richer food resource for the carnivorous species *Cyclops* sp., and under the regulation of predation, it further promotes the growth of copepods. With the increase in *Cyclops* sp., the food chain is extended, and more predator–prey relationships are formed, offering more ecological niches and interactions. Thus, the network of biological interactions is developing in a more complex direction (Jiang and Pu 2009; Sweeney et al. 2022). Longer food chains usually mean that more species in the ecosystem are involved in the flow of energy and matter, increasing the species diversity of the community and thus improving the stability of the ecosystem as well as its resistance to perturbations (Zhao et al. 2023). In addition, high‐temperature domestication increases the maximum predation rate of predators. This improvement in predation efficiency may indirectly promote the elongation of food chains by enhancing the strength and frequency of predator–prey relationships. Such food chains contain more predator–prey relationships, provide more ecological niches and interspecific interactions, and ultimately render the biological interaction networks during the heatwave taming period more complex. (Sentis et al. 2015).

In addition, although biomass may decrease during high‐temperature heat waves, aquatic ecosystems may be stabilized to a certain extent by altering the structure of the food web (e.g., by changing the strength of interactions between species) (Brooks and Dodson 1965). On the other hand, the average degree and clustering coefficient of the phytoplankton–zooplankton interactions network expanded with the development of a sustained heatwave, as well as with the development of heatwave to a later stage, suggesting that the structure of the network may recover after an extreme event (Polazzo et al. 2008).

Within a certain range, ecosystems may recover to their initial state after being disturbed (Shade et al. 2012). Heatwaves act primarily as impulse perturbations in many laboratory studies (Nascimento et al. 2024). Recent research on the effects of pulse perturbations on community stability suggests that the functional effects of communities may initially be severely affected by pulse perturbations, but functioning usually recovers within the time of the experiment (Hillebrand and Kunze 2020). Although compositional stability may be altered by heatwaves, functional stability may be maintained by adjustments within the ecosystem (Pérez et al. 2022). Such adjustments may involve reorganization of species interactions and redistribution of energy flows (Konopka 2009). The network remained highly connected and complex in the post‐heatwave period, which deserves further studies. We speculate that the large proliferation of nauplius during the heatwave taming period could be used as a reserve biomass in the post‐heatwave period along with the large number of maturing and developing adults in the post‐heatwave period as a way to maintain the stability and complexity of the network (Nascimento et al. 2024).

### 4.4 Impacts of Sustained Heatwaves on Aquatic Ecosystem

Sustained heatwaves may alter key eco‐physiological processes of aquatic ecosystem organisms, such as respiration, nutrient uptake and metabolic rates, affecting the photosynthetic efficiency of primary producers and inducing predator stagnation (Huang et al. 2021; Jueterbock et al. 2016). In our study, phytoplankton, representing primary producers, and zooplankton, representing primary consumers, demonstrated consistent trends in cell abundance changes, with reduced phytoplankton cell abundance in the heatwave period and the heatwave taming period, which were subjected to heatwave stress.

The plankton community structure of the water column was also altered by the heatwave, with the dominance of phytoplankton decreasing during the heatwave taming period, while the relative abundance of benthic diatoms increased, and the abundance of the carnivorous Copepoda *Cyclops* sp., both benthic and planktonic, rose. This indicates a shift from a community structure dominated by phytoplankton to one containing more benthic components. The ecological niche differentiation of species may lead to an increase in functional diversity in the community and a decrease in competition between planktonic organisms to improve resource use efficiency, which in turn increases the complexity and stability of the aquatic food web (Kelly et al. 2021).

Larger‐sized phytoplankton blooms result in the dominance of herbivores in the aquatic food web (Legendre and Rassoulzadegan 1995). In contrast, microzooplankton feed primarily on smaller phytoplankton species (Agasild et al. 2007). In our study, although the relative abundance of phytoplankton was lower in the heatwave taming period, an increase in larger‐bodied phytoplankton dominant species, such as *Aulacoseira granulata* , was able to feed zooplankton with larger monomodal biomasses (a shift from a Rotifera‐dominated zooplankton community to one dominated by Copepoda and Cladocera). Larger‐bodied zooplankton may have higher biomass and energy densities, possibly because they are able to support more energy demand per unit of biomass and are more efficient in energy utilization (Pyrina and Domeisen 2023).

A key issue in ecology is quantifying the relative roles of deterministic and stochastic processes in biological communities (Chai et al. 2016). Deterministic and stochastic processes can simultaneously regulate the structure of biological communities (Yu et al. 2019). The present study indicates that water temperature, turbidity, conductivity, water depth, and ammonia nitrogen were significantly correlated with zooplankton, findings that have been previously reported in shallow subtropical lakes (Guo et al. 2023; Zhang et al. 2016). In general ecological studies, heatwave events are often considered stochastic processes; however a sustained heatwave may gradually be recognized as a more deterministic process in ecosystems (Russo 2015; Shafiei Shiva and Chandler 2020). Heatwaves may have disruptive effects on some components of aquatic ecosystems, especially for species that have long been adapted to deterministic processes (Ainsworth et al. 2020; Guo et al. 2024). This disruption may lead to long‐term changes in ecosystem functioning and may even trigger ecosystem transformations (Zhou et al. 2023). During sustained heatwave events, species in the ecosystem may adapt to new environmental conditions through evolution or domestication, such as high‐temperature acclimatization in Copepoda (Broglio et al. 2003).

With global climate change, some regions may experience more frequent and intense heatwave events, which may shift from being stochastic processes to more common and persistent phenomena in ecosystems, but do not imply a loss of stochasticity (Zhou et al. 2023). Rather, they may become a more significant and persistent factor in ecosystem dynamics, and the results of this study provide new perspectives on water ecosystem management and conservation.

## Author Contributions

**Lingli Jiang:** conceptualization (lead), investigation (equal), methodology (lead), visualization (lead), writing – original draft (lead), writing – review and editing (equal). **Mengfan Sun:** investigation (equal). **Zhongze Zhou:** writing – review and editing (equal). **Yutao Wang:** writing – review and editing (equal).

## Conflicts of Interest

The authors declare no conflicts of interest.

## Supporting information


**Data S1:** Table of water quality and plankton data.

## Data Availability

The data that support the findings of this study are available in Data S1 of this article.
